# Notes on the Recent Progress in Ophthalmology

**Published:** 1895-12

**Authors:** Frank C. Todd

**Affiliations:** Fort Worth, Texas


### Notes on the Recent Progress in Ophthalmology.

BY FRANK C. TODD, M.D., FORT WORTH, TEXAS.

RIPENING OF IMMATURE CATARACT BY DIRECT TRITURATION.—Bettman (*Annals of Ophth. and Otol., IV., No. 1*) gives a short history of the operations for ripening cataracts preparatory for removal. He mentions Forster's operation as being the method most frequently employed. The principle involved is to break down the lens fibres, and thus induce their rapid degeneration and opacity. This is accomplished by pressing on the cornea, after doing an iridectomy, with a spoon or strabismus hook. The force is transmitted indirectly to the unripe cataract, and through this massage it is rendered entirely opaque in a varying period of time.

The objections to this method were the necessity of doing iridectomy, and the danger of setting up an iritis from the mechanical irritation.

White modified the operation, and did away with the necessity of doing an iridectomy, by first making a paracentesis, and drawing off the aqueous humor, thereby permitting the lens to come into contact with the posterior surface of the cornea, and then making massage.

Bettman claims that the best results may be obtained by practicing his operation, which he describes as follows:

After entering the anterior chamber with a small lance-shaped knife, a trowel-shaped spatula is introduced, its flat surface placed against the lens, either in the pupillary area, or when the iris is contracted, underneath the iris, and a half dozen to a dozen passes made over the cataractous lens. The pressure exerted is uniform, and not severe. The spatula is then withdrawn, the eye washed with antiseptics, and bandaged never longer than two days. Ordinarily one day. No iridectomy is done.

He never performs the operation upon sclerosed, amber-colored cataracts, such as never become entirely opaque.

He has operated upon cases successfully when the cataract was only so far advanced that the fundus can be seen.

His conclusions are:

(1) Artificial ripening of cataracts is, in properly selected cases, demanded; (2) direct trituration is preferable to other methods,—it is easily performed by one possessing ordinary skill; (3) it is not followed by untoward symptoms, consequently it is a safe and reliable procedure; (4) it is not indicated where sclerosis involves the bulk of the lens; (5) it is especially useful in senile cataracts with soft cortex; (6) the results of the massage are marked and rapid; (7) maturity of the cataract is usually induced in three weeks, often sooner; (8) very little discomfort is caused the patient aside from the bandaging the eye two days; (9) at the subsequent extraction of the lens, the cortical substance is readily removed, and dangers of iritis and suppuration of the corneal wounds are lessened.

---

THE ACTION OF THE OBLIQUE MUSCLES IN ASTIGMATISM.—

Wilson (*Archiv. of Oph., XXIII., No. 3*) draws these conclusions: 1. Compensatory rotation of the eyeballs in astigmatism will be necessary in order to secure binocular vision, whenever the predominant diffusion lines of any object upon the retina of one eye and the normal or diffusion-line image of the other eye do not fall upon corresponding retinal points. 2. This lack of retinal correspondence, or rotating of the image, is a function of (*a*) the amount and character of the astigmatism; (*b*) the nature of the ametropia; (*c*) the nature of the object viewed; (*d*) the action of ciliary muscles.

---

LID PRESSURE AS A CAUSE OF ASTIGMATISM.—Howe (*Amer. Journal of Ophth.*, Sept., 1894) has demonstrated on himself and others the fact that corneal astigmatism can be produced by persistent strong lid pressure in half-closing the eye at their work, an average astigmatism of 0.905 D.

---

THE PRACTICAL VALUE OF LOW-GRADE CYLINDERS IN SOME CASES OF ASTHENOPIA.—White (Ibid, 1894) finds from considerable experience the value of correcting small degree of astigmatism, even as low as 0.25 D, in relieving asthenopia, particularly when the axis was off the horizonal or perpendicular, or when anisometropia was present.

---

EPITHELIOMA SIMULATING ULCERATED MEIBOMIAN CYST.—De Sweinitz (Trans. Amer. Ophth. Soc., 1894) had a case in which a large granular mass protruded from what appeared to be a chalazion of the upper lid. It was excised and the cavity curetted. Examination showed it to be epitheliomatous.

---

THE STATISTICS OF TRACHOMA.—Van Millingen (*Annales D'Oculistique CXIV, No. 3*) gathered statistics from representative ophthalmologists all over the world, and draws the following conclusions:

1. Trachoma is an infectious and contagious disease which predominates in uncivilized countries and tends to disappear with the progress of civilization and of hygiene. Hygiene and cleanliness are the best preservatives against trachoma.

2. Trachoma is not influenced by altitude; it may spread wherever the people are uncleanly and live in poverty, quite as easily at altitudes of from 1,000 to 5,000 metres as on plains.

3. All races are equally susceptible to the virus of trachoma. An immunity for certain races does not exist.

THE INDICATIONS FOR, AND THE ADVANTAGES AND TECHNIQUE OF, MUSCLE SHORTENING.—Savage (*Ophth. Record, V, No. 3*) believes that muscle shortening is indicated in all cases of heterophoria regardless of the direction or extent of the turning. He stated that shortening alone would, in many cases, effect a complete cure of the squint, while in many other cases the shortening should be associated with a partial tenotomy of the stronger muscle. In all cases of heterophoria not cured by a partial tenotomy done but one time on any one muscle, the remainder should be corrected by shortening the weaker muscle or by rhythinic exercise when possible.

The advantages he claims for shortening a muscle over an advancement, as formerly practiced, consists of the great ease with which the former operation is done, and the great comparative freedom from danger of effecting torsion of the eye, which is so likely to occur in an advancement; and if the knot should become untied in the shortening operation, the patient's condition would not be worse than before.

---

THE EFFICACY OF APPLICATIONS OF TINCTURE OF IODINE IN THE TREATMENT OF INFECTIOUS ULCERS OF THE CORNEA.—Van den Bergh (*An. D. Oc. from the Presse Médic, Belge*, Aug., 1895) reports a case of ulcer of cornea, with hypopyon, treated and cured in eight days by application of iodine tincture, on a cotton tipped probe, at first twice a day and afterwards once daily, carrying out other treatment as demanded. He thinks this method to be the surest and simplest method and at the disposal of every general practitioner. Whereas the management of the cautery necessitates considerable practice.

If by chance the application slightly overlaps the limits of the ulcer, there will be only a slight desquamation of the corneal epithelium, which will be restored without leaving a trace.

---

A CASE ILLUSTRATING THE BAD EFFECTS WHICH THE ESTABLISHMENT OF MENSTRUATION MAY HAVE UPON THE COURSE OF INTERSTITIAL KERITITIS.—Dunn (*Archives of Opth., XXIV, No. 3*) reports the case of a fourteen year old girl, whom he was treating for specific keritities, which improved under constitutional treatment. The photophobia, however, remained intense, and was worst when she went to bed March 18. *When she awoke on the next morning the photophobia had entirely disappeared and with it practically all evidences of active inflammation of the cornea.* Menstruation had become established during the night.

The damage done to the sight was beyond hope of repair. Both cornea were cloudy and numerous enlarged lymph channels and blood vessels had made their appearance.

---

EXPERIMENTAL INVESTIGATION OF PATHOGENESIC OF SYMPATHETIC OPHTHALMIA.—Bach (*Ann. D'Ocul., XIV, No. 3*) made experiments with staphylococcis of different virulence and with the bacilli of tuberculosis. He was not able to verify a passage from one eye to another by means of the optic sheaths.

Furthermore, after enucleations performed for sympathetic ophthalmia, either in the course of or at the beginning of the disease, bacteriological examinations of the nerve or of the organ itself were negative.

On the other hand, any stimulation whatever of the ciliary nerves of one eye, was followed in the other eye by disturbances appreciable on microscopic examinations. These consisted of a fibrinous excretion collected in multiple foci and of extravasation of blood corpuscles. The seat of these lesions was the anterior portion of the vitreous humor, the ciliary walls, the anterior and posterior chambers. Here and there were formed swellings of the epithelium of the ciliary processes, and in the vesicles thus formed was a fibrinous precipitate.

The author thinks that the coagula of fibrin were the initial lesions in the sympathetic process.

Irritation from the sympathizing eye will follow the centripetal canals; the ciliary nerves, the ciliary ganglion, especially the sympathetic root of the carotid plexus of the great sympathetic; from thence, by the circle of Willis, it will travel to the sympathetic of the other side to reach the sympathized eye by the centrifugal canal of the ciliary nerves, and especially by the vascular nerves.

---

EPHEDRIN HOMATROPINE.—Super (*N. Y. Med. Jour. LXI. No. 23*) uses this combination in the following proportion:

| | |
|---|---|
| Ephedrin hydrochlor.......... | 1.00 |
| Homatropine hydrochlor....... | 0.01 |
| Aq. dist...................... | 10.00 |

He uses it merely as a mydriatic for diagnostic purposes, for it is more rapid in its action than other drugs, is sufficiently intense and does not affect the accommodation.

The full mydriasis lasts a half hour and the pupil acquires its normal size in from four to six hours.

This combination is also put up under the name of *mydrin*.

GROWTH OF THE EYE.—Weiss, (*Annales D'Oculestique CXIV No. 3*). Measurements made on the eyes of the new-born and of adult show that the volume of the eye increases three or four times, while the volume of the entire body increases in proportion of 1:21. Curves show that the increase in the eye is proportionate to the increase in the volume of the brain. All diameters of the eye increase symmetrically.

---

MEDICAL TREATMENT OF GLAUCOMA.—Groenouw and Dufour (*An. D'Oc. CXIV No. 3*). Glaucoma improves in tension for years by continued use of eserine and pilocarpine. The visual field, however, is not modified, and excavation of the disc did not cease to progress. Strong light frequently acts in glaucomatous persons, as a myotic, by contracting the pupil. Dufour has seen cases of glaucoma exaggerated by the influence of darkness.

